# Investigation of the piroplasm diversity circulating in wildlife and cattle of the greater Kafue ecosystem, Zambia

**DOI:** 10.1186/s13071-020-04475-7

**Published:** 2020-11-30

**Authors:** David Squarre, Yukiko Nakamura, Kyoko Hayashida, Naoko Kawai, Herman Chambaro, Boniface Namangala, Chihiro Sugimoto, Junya Yamagishi

**Affiliations:** 1 Research Center for Zoonosis Control, Hokkaido University, Sapporo, Japan; 2 Wildlife Veterinary Unit, Department of National Parks and Wildlife, Chilanga, Zambia; 3 The Royal (Dick) School of Veterinary Studies, University of Edinburgh, Edinburgh, UK; 4 Central Veterinary Research Institute, Ministry of Fisheries and Livestock, Chilanga, Zambia; 5 Department of Paraclinical Studies, University of Zambia, Lusaka, Zambia; 6 International Collaboration Unit, Research Center for Zoonosis Control, Hokkaido University, Sapporo, Japan

**Keywords:** *Piroplasma*, Meta-barcoding, Kafue ecosystem, Zambia

## Abstract

**Background:**

Piroplasms are vector-borne intracellular hemoprotozoan parasites that infect wildlife and livestock. Wildlife species are reservoir hosts to a diversity of piroplasms and play an important role in the circulation, maintenance and evolution of these parasites. The potential for likely spillover of both pathogenic and non-pathogenic piroplasm parasites from wildlife to livestock is underlined when a common ecological niche is shared in the presence of a competent vector.

**Method:**

To investigate piroplasm diversity in wildlife and the cattle population of the greater Kafue ecosystem, we utilized PCR to amplify the 18S rRNA V4 hyper-variable region and meta-barcoding strategy using the Illumina MiSeq sequencing platform and amplicon sequence variant (ASV)-based bioinformatics pipeline to generate high-resolution data that discriminate sequences down to a single nucleotide difference.

**Results:**

A parasite community of 45 ASVs corresponding to 23 species consisting of 4 genera of *Babesia*, *Theileria*, *Hepatozoon* and *Colpodella*, were identified in wildlife and the cattle population from the study area. *Theileria* species were detected in buffalo, impala, hartebeest, sable antelope, sitatunga, wild dog and cattle. In contrast, *Babesia* species were only observed in cattle and wild dog. Our results demonstrate possible spillover of these hemoprotozoan parasites from wildlife, especially buffalo, to the cattle population in the wildlife-livestock interface.

**Conclusion:**

We demonstrated that the deep amplicon sequencing of the 18S rRNA V4 hyper-variable region for wildlife was informative. Our results illustrated the diversity of piroplasma and the specificity of their hosts. They led us to speculate a possible ecological cycle including transmission from wildlife to domestic animals in the greater Kafue ecosystem. Thus, this approach may contribute to the establishment of appropriate disease control strategies in wildlife-livestock interface areas.
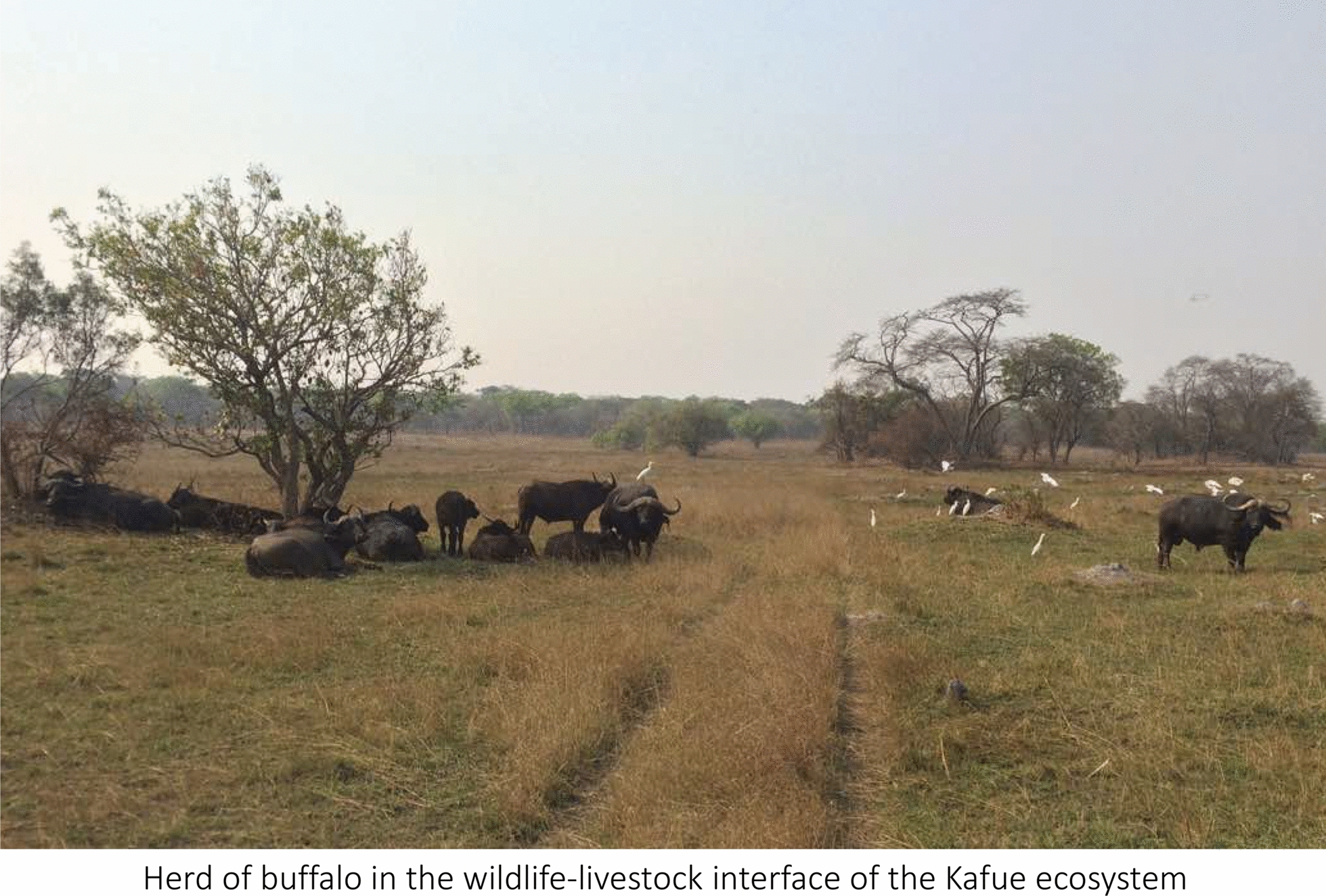

## Background

Piroplasmida is an order of intracellular heamoprotozoan parasites that belong to the phylum Apicomplexa. The species of genera *Theileria* and *Babesia* cause clinical disease in vertebrate hosts including domestic and wild animals [1, 2]. The parasites are transmitted by vectors of ixodid ticks and have a considerable socio-economic impact on livestock production in sub-Saharan Africa, threatening livelihoods and food security [3]. The *Theileria* and *Babesia* genera consist of a wide diversity of species and genotypes [4, 5].

Wildlife plays an important role in the circulation, maintenance and evolution of these parasites. African buffalos (*Syncerus caffer*), for example, are reservoirs of buffalo-derived *Theileria parva*, which causes theileriosis or corridor disease in cattle [6, 7]. This disease is transmitted from buffalo to cattle and not between cattle, because cattle acutely die before piroplasms emerge or infect new ticks [8, 9]. Conversely, in East Coast fever (ECF) caused by *T. parva* circulating among the cattle population, some infected cattle survive because of the immune response and occasional chemotherapy and then become asymptomatic carriers, leading to the continuous spread of the parasite among cattle [10].

Although *Theileria* is by far the most important piroplasma having a considerable effect on livestock production, *Babesia* also cause a wide range of infectious diseases in domestic animals. Redwater in cattle, canine babesiosis and equine piroplasmosis are caused by *Babesia bigemina/B. bovis*, *B. canis* and *B. caballi/B. equi*, respectively. Several wildlife species are natural hosts of a wide diversity of piroplasma that are either pathogenic or non-pathogenic to domestic animals.

The greater Kafue ecosystem, measuring 68,000 km^2^ in size, is a large conservation area in central Zambia. It is composed of the Kafue National Park (22,400 km^2^) and nine adjacent game management area (GMAs) that act as a buffer to the national park. The national park is host to numerous wildlife species and particularly is devoid of human settlements and livestock. The GMAs that immediately surround the park are notably characteristic of wildlife cohabiting with communities and their livestock, thus forming a wildlife-livestock interface area [11, 12]. The potential for likely spillover of arthropod-borne pathogens such as piroplasmas from wildlife to livestock occurs when a common ecological niche is shared in the presence of a competent vector [13]. In addition to the interface in conservation areas, the growing game ranching industry in Zambia has integrated wildlife and livestock farming, creating widespread patches of ex-situ wildlife-livestock interface areas across the country. The primary source of wildlife for stocking game ranches is conservation areas such as the greater Kafue ecosystem. This is likely to spread parasites and create a vortex of piroplasm parasites across the country.

Highlighting comprehensive piroplasm parasite community composition including cryptic species/genotype diversity of circulating parasites in the wildlife, livestock and vector population is essential to understand disease ecology and to prepare optimized countermeasures. A reservoir of important pathogens and their transmission path will be illustrated by this approach. Interaction between pathogens under mixed infection, which may cause discriminated manifestation, is another interest. Understanding the parasite community also has implications for the choice of assays to adopt in control options such as calf immunization and in epidemiology studies of piroplasm infections [14–18]. It provides basic information for the selection of live or recombinant vaccines to be used in a specific area as well [19].

To investigate the parasite diversity, deep amplicon sequencing of the 18S rRNA V4 hyper-variable region by next-generation sequencing (NGS) technology has been developed [14, 17, 20, 21]. The scheme has been adopted for cattle [20], African buffalo [14, 17], Asian buffalo, cattle and sheep [21]. We also adopted the scheme and expanded the target to whole wildlife in this study to investigate and illustrate the diversity of the piroplasm community in wildlife and cattle in a discrete geographical region of the greater Kafue ecosystem of Zambia.

## Methods

### Sample collection and DNA extraction

**Fig. 1:**
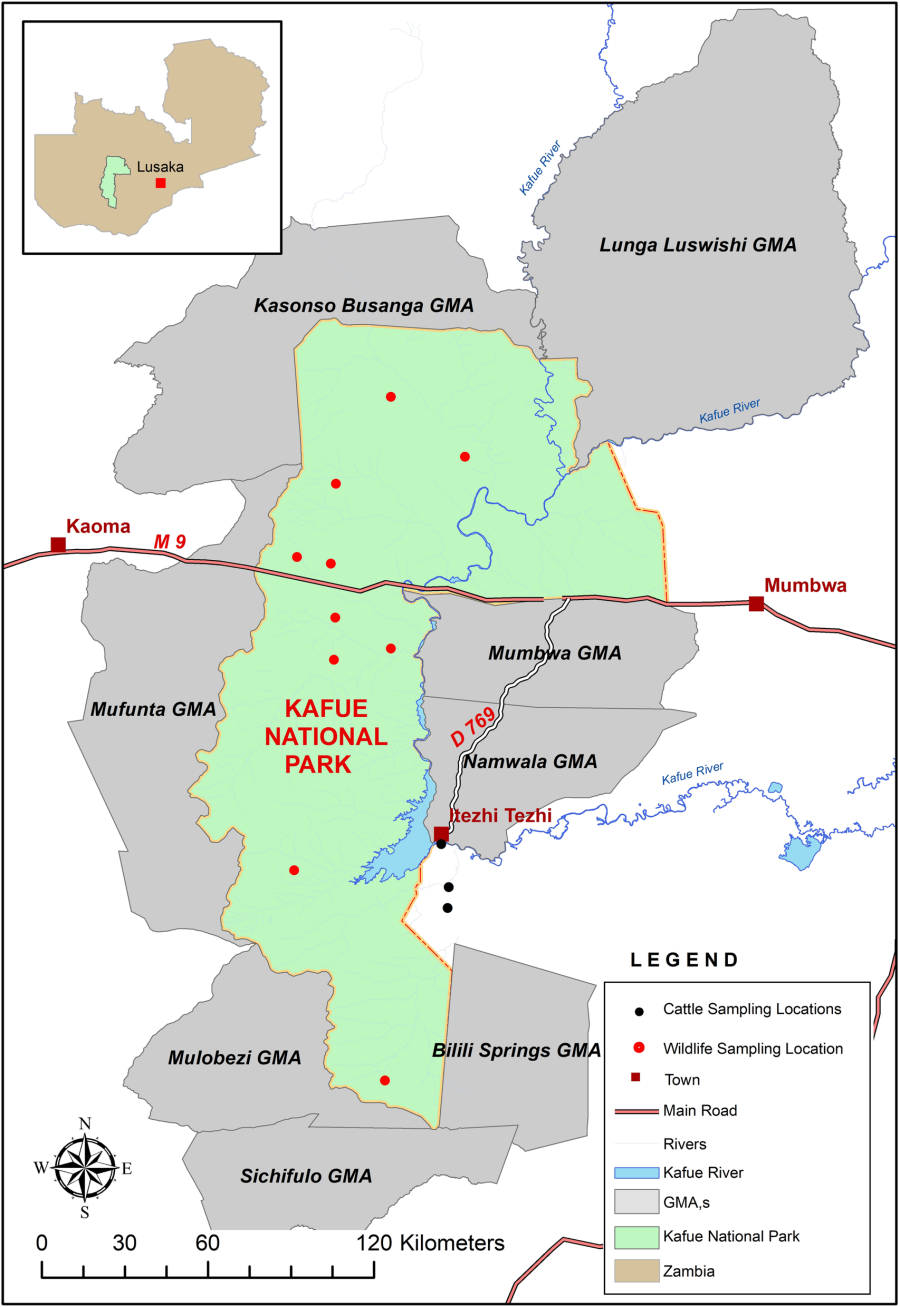
Map of the Kafue ecosystem consisting of the Kafue National Park and the game management areas (GMAs) showing sampling sites of wildlife and cattle

The greater Kafue ecosystem (Fig. 1) is a conservation area located in central Zambia (14°03″ S/16°43″ S and 25°13″ E/26°46″ E) measuring about 67,806 km^2^ in size. Whole-blood samples were collected from wild animals captured during a restocking program conducted between May and August of 2017 and 2018. Approximately 5 mL of blood was collected through venipuncture into EDTA vacutainers and immediately placed on ice. The samples were collected from 253 wild animals consisting of 12 wildlife species (Table 1) during chemical immobilization and physical restraint as previously described [22, 23]. An additional 232 blood samples were collected from cattle in the interface between the GMA and open area in Zambia’s Itezhi-Tezhi district between April and May 2019 (Table 1).

**Table 1:** Detection of heamoparasites in wildlife species and cattle from the Kafue ecosystem using RLB-PCR

	Species sampled	Number	RLB-PCR positive	Positive rate (%)
1	Impala (*Aepyceros melampus*)	106	65	61.3
2	Hartebeest (*Alcelaphus buselaphus*)	47	19	40.0
3	Sable antelope (*Hippotragus niger*)	8	8	100
4	Lion (*Panthera leo*)	4	1	25.0
5	Wild dog (*Lycaon pictus*)	2	2	100
6	Sitatunga (*Tragelaphus spekii*)	4	3	74.0
7	Buffalo (*Syncerus caffer*)	53	33	62.3
8	Lechwe (*Kobus leche leche*)	9	0	0.0
9	Cheetah (*Acinonyx jubatus*)	1	0	0.0
10	Vevert monkey (*Chlorocebus pygerythrus*)	1	0	0.0
11	Baboon (*Papio ursinus*)	1	0	0.0
12	Warthog (*Phacochoerus africanus*)	17	0	0.0
13	Cattle (*Bos taurus*)	232	147	63.4
	Total	483	278	57.6

From each blood sample collected, genomic DNA was extracted using the DNA Isolation Kit for Mammalian Blood (Roche Applied Science, Indianapolis, IN, USA) for wild animal samples and QuickGene DNA Whole-Blood Kit S (Kurabo, Osaka, Japan) for cattle samples as per the manufacturer’s protocol. A final volume of 200 μL DNA was eluted in tubes and stored at − 80 °C until analysis.

### RLB-PCR amplification and library preparation

Amplification of the V4 hypervariable region of the 18S rRNA gene was obtained by piroplasma-specific RLB-PCR using primers RLB-F and RLB-R (Table 2) [24]. The 10 μL reaction mix contained 5.0 μL Ampdirect plus buffer (Shimadzu, Kyoto, Japan), 3.95 μL PCR-grade water, 0.05 μL Bio Taq HS (Bioline, London, UK), 0.5 μL DNA template and 0.25 μL each of the RLB primers. The thermocycler conditions were 94 °C for 10 min denaturation and 40 cycles of 94 °C for 1 min, annealing at 50 °C for 1 min, extension at 72 °C for 1.5 min and final extension at 72 °C for 10 min.

**Table 2:** Primers used for piroplasm parasite detection

Primer’s target region	Primer name	Primer sequence (5′–3′)	References
18S rRNA V4 hyper-variable region	Reverse Line Blot-F (RLB-F)	GAGGTAGTGACAAGAAATAACAATA	[24]
	Reverse Line Blot-R (RLB-R)	TCTTCGATCCCCTAACTTTC	
Illumine tail-tagged RLB primer	Illumina RLB-F	ACACTCTTTCCCTACACGACGCTCTTCCGATCT[RLB-F]	
	Illumina RLB-R	GTGACTGGAGTTCAGACGTGTGCTCTTCCGATCT[RLB-R]	
Illumina-index primer	Illumina-i5 primers	AATGATACGGCGACCACCGAGATCTACAC[index*] ACACTCTTTCCCTACACGACGCTCTTCCGATCT	
	Illumina-i7 primers	CAAGCAGAAGACGGCATACGAGAT[index*] GTGACTGGAGTTCAGACGTGTGCTCTTCCGATCT	

^*^Index: 8-bp nucleotide to provide a unique index to each sample

The second PCR adding Illumina tail was conducted using 100 times diluted first PCR amplicons as template. The reaction volume of 10 μL comprised the same volumes of reagents as the first RLB-PCR but instead replaced the primer with 10 μM Illumina tail-tagged RLB primers (Table 2). The thermocycler conditions were the same as for the first PCR except amplification was set at 12 cycles.

The Illumina tail-tagged amplicons from the second PCR were then diluted 50 times, and 1 μL was added to a 20 μL reaction mixture for index PCR. The other reagents included 4 μL of 5× buffer, 1.4 μL MgCl_2_ (25 mM), 0.5 μL 10 mM dNTP mix, 1 μL mixed Illumina-index primer (Table 2), 11.975 μL nuclease-free water and 0.125 μL KAPATaq Extra. The indexing PCR was run with thermocycler conditions of 95 °C initial denaturation for 5 min followed by 15 cycles of 92 °C for 30 s, 45 °C for 30 s and 72 °C for 30 s and a final extension at 72 °C for 15 min. The obtained amplicons, which have a unique 8-bp index on both sides for each sample, were quantified by agarose gel electrophoresis. Then, an equal amount of each sample was pooled into one library and gel-purified using Wizard SV Gel and the PCR Clean-Up System (Promega, Madison, WI, USA).

### Amplicon sequencing and bioinformatic analysis

The RLB-PCR amplicon library was sequenced with the MiSeq [17, 21] using a 300-bp paired-end sequencing protocol and the MiSeq Sequencing Reagent Kit v3 (Illumina, Hayward, CA, USA) with 25% PhiX DNA spike-in control according to the manufacturer’s instructions. Quality control and filtering were conducted with Trimmomatic [25] using the following parameters: TRAILING:20, SLIDINGWINDOW:4:15 and MINLEN:36. Concatenation between forward and reverse reads and primer trimming was conducted with AMPtk [26], allowing a minimum merged length of 400 bp. Primer sequences to be trimmed were GAGGTAGTGACAAGAAATAACAATA and TCTTCGATCCCCTAACTTTC for forward and reverse reads, respectively.

A set of amplicon sequence variants (ASVs) was generated by DADA2 and LULU in the AMPtk package using the default parameters. The obtained sequences were annotated based on sequence homology with the Basic Local Alignment Tool (BLAST) and non-redundant nucleotide database by NCBI using -max_target_seqs 1, -perc_identity 70, -qcov_hsp_perc 70 and -evalue 1e-20 as a set of parameters [27]. Operational taxonomic units (OTUs) were further generated by clustering the ASVs using usearch [28] with 99% identity as clustering threshold. Observed ASVs in each sample were filtered out if the number of the assigned reads was < 1% of the total number of assigned reads.

### Phylogenetic analyses

The phylogenetic relationships among ASVs were analyzed using the neighbor-joining method [29] implemented in MEGA X [30]. The evolutionary distances were computed using the maximum composite likelihood method [31] and default parameters with 10,000 bootstraps. Visualization and annotation were conducted using iTOL v5.5 [32]. Each clade was annotated based on sequence identity obtained by the BLAST analysis.

## Results

### Detection of the piroplasm parasite by PCR and taxonomical annotation

Of 253 sampled wild animals, 61.3% (65/106) of impalas, 40% (19/47) of hartebeests, 62.3% (33/53) of buffalos, 74% (3/4) of sitatungas, 25% (1/4) of lions and all the sable antelope (8/8) and wild dogs (2/2) were positive for piroplasmosis by RLB-PCR. In case of cattle, 63.4% (147/232) were RLB-PCR positive. Of the 12 wildlife species sampled and screened, 7 species were infected by piroplasm parasites (Table 1, Additional file 1: Table S1). All the positive amplicons were subjected to sequence analysis to identify their taxonomic classification. In total, 2.80 M raw reads were obtained from 278 PCR positive samples and then merged into 2.46 M contigs (Additional file 1: Table S1).

A total of 45 ASVs of the V4 hyper-variable region of the 18S rRNA gene were obtained from both wildlife species and cattle sampled from the greater Kafue ecosystem (Table 3). The taxonomic assignment of ASV using BLASTn resulted in the identification of four genera, *Theileria*, *Babesia*, *Hepatozoon* and *Colpodella*, which consisted of 11, 3, 2 and 1 known species and 36, 6, 2 and 1 ASVs, respectively (Additional file 1: Table S1).

**Table 3:** Diversity of piroplasmas detected in wildlife and cattle samples collected from the Kafue ecosystem

Genus	Species	Genotypes	BLAST top hit	ID	OTU	ASV	% Identity
*Theileria*	*T. velifera*	*T. velifera*	*T. velifera* KSA_D_Th6	LC431550	1	*1*	100.00
						7	99.78
						64	99.78
						92	99.78
		*T. velifera* A	*Theileria cf. velifera* A	GU733375	2	6	98.69
		*T. velifera* B	*Theileria cf. velifera *(*Syncerus caffer*) clone H4a	JN572701	1	*29*	100.00
						55	99.78
	*T. mutans*	*T. mutans*	*Theileria mutans* isolate MT15	KU206320	11	*3*	100.00
						16	99.78
		*T. mutans* MSD	*Theileria* sp. B15a	JN572700	12	*5*	100.00
		*T. mutans*-like 1	*Theileria cf. mutans* 1 (*Syncerus caffer*) clone C21b	JN572699	13	26	99.56
		*T. mutans*-like 2	*Theileria cf. mutans* 2 (*Syncerus caffer*) clone Q15d	JN572696	14	42	98.68
						60	98.46
	*T. parva*		*Theileria parva* isolate F45P16	MH929322	23	*15*	100.00
			*Theileria parva*	AF013418	23	86	99.78
						101	99.56
	*Theileria *sp. (buffalo)		*Theileria* sp. ex *Syncerus caffer* MCO-2011 clone V8b	HQ895982	23	*10*	100.00
						11	99.78
						18	99.56
						22	99.56
	*Theileria *sp. (bougasvlei)		*Theileria* sp. KS-2015 isolate CAT79	KP410267	22	*25*	100.00
	*T. taurotragi*		*Theileria taurotragi*	L19082	20	35	97.80
					21	46	99.78
						*62*	100.00
	*Theileria* sp. (sable)		*Theileria* sp. BM-2010/sable	GU733378	16	8	99.78
						14	99.57
						85	99.35
	*Theileria* sp. (waterbuck)		*Theileria* sp. NG-2013c isolate waterbuck 39 clone 6	KF597072	19	4	99.35
	*Theileria* sp. (tsessebe)		*Theileria* sp. ex Damaliscus lunatus clone TS22_11	HQ179766	16	9	99.57
						23	99.78
						*49*	100.00
	*Theileria* sp. (giraffe)		*Theileria* sp. NG-2012b isolate 44 clone 2	JQ928925	15	2*	95.90
						106*	95.66
						47*	95.71
	*Theileria *sp. (bongo)		Uncultured *Theileria* sp. isolate BNG13	MH569462	17	12	97.82
			Uncultured *Theileria* sp. isolate BNG10	MH569463	18	13	99.57
*Babesia*	*B. bigemina*		*Babesia bigemina* clone PR28CL7	MH050387	7	32	100.00
						71	99.77
			*Babesia bigemina* isolate B_bi11	EF458200	7	67	100.00
			*Babesia bigemina* clone PR38CL1BBIG	MH047819	7	19	100.00
	*Babesia* sp.		*Babesia* sp. 9 1093 cl1	KX218437	10	58	98.95
	*B. occultans*		*Babesia occultans* isolate Trender1	KP745626	6	28	100.00
*Hepatozoon*	*H. canis*		*Hepatozoon canis* isolate 70	MK645969	8	40	99.60
	*Hepatozoon* sp.		*Hepatozoon* sp. 2 BCS-2013 isolate L4	KF270665	9	82	99.00
*Colpodellidae*			Uncultured *Colpodellidae* clone PL31	MN103986	3	57	100.00

ASV numbers shown in italics and with an asterisk represent the identity against the reference sequence of 100% and 95.7–95.9%, respectively

*OTU* operational taxonomic unit, *ASV* amplicon sequence variant

**Fig. 2:**
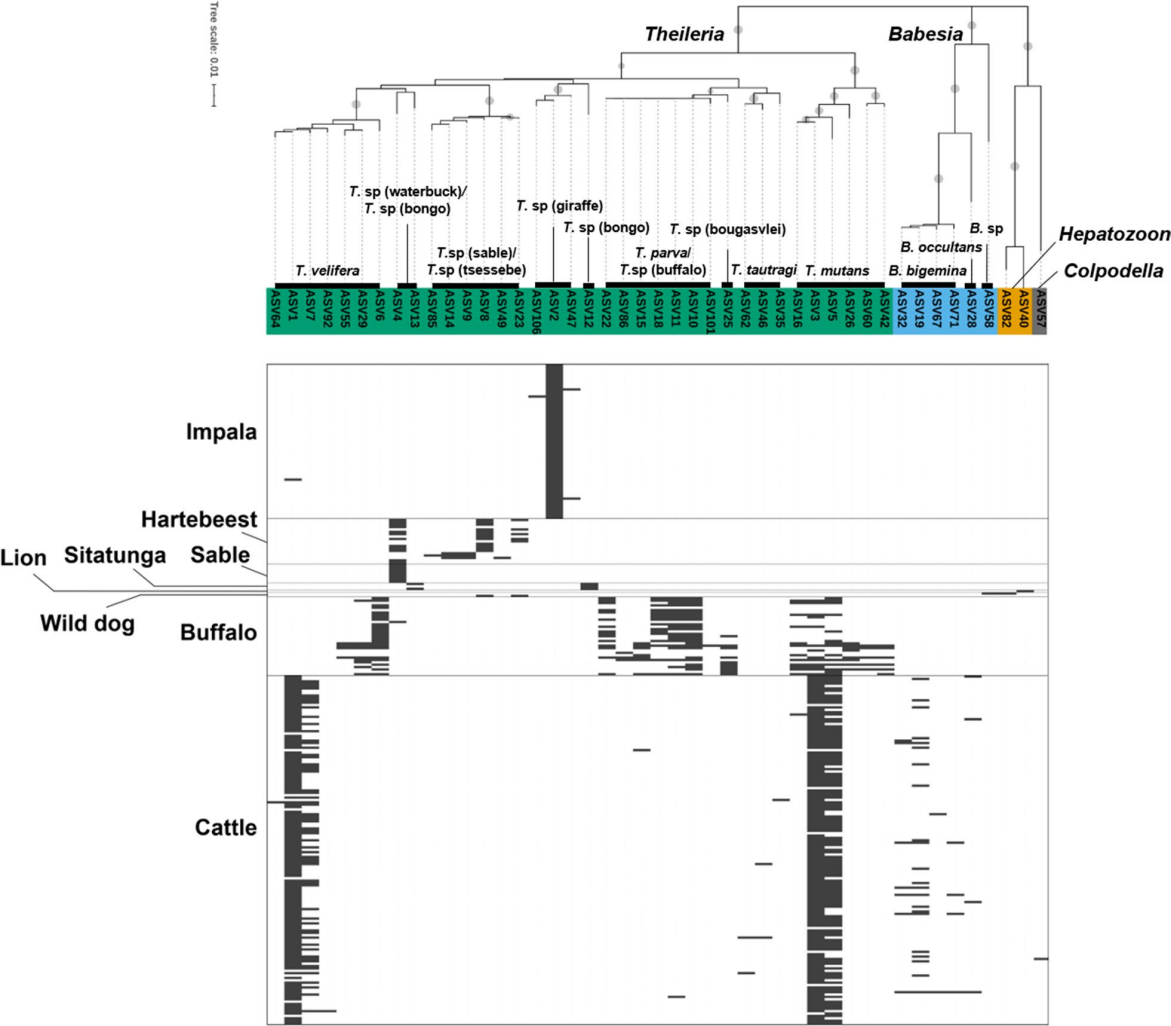
Phylogenetic tree of ASVs and the map of positive ASVs per animal species. On the top is the neighbor-joining tree of 45 ASV sequences, using a total of 411 positions in the final dataset. Bootstrap values > 70 are shown as proportionate size circles for each node. The tree is drawn to scale, with branch lengths in the same units as those of the evolutionary distances used to infer the phylogenetic tree. Below is a table showing the positive wildlife and cattle samples for each ASV

In the phylogenetic analysis, we observed both *Theileria* and *Babesia* clade (Fig. 2). The *Theileria* clade consisted of a subclade for *T. velifera*, *T. mutans*, *T. parva* and *T. taurotragi*.

The *T. velifera* subclade consisted of seven ASVs, and sequence identity to *T. velifera* was 98.7% to 100% (Table 3), suggesting all of these ASVs belong to *T. velifera.* The subclade was further divided into two groups based on sequence identity. One was ASV6, 29 and 55, which were detected only in buffalo; ASV7, 64 and 92 were detected only in cattle while ASV1 was detected in cattle and impala (Fig. 2, Additional file 1: Table S1).

A similar correlation among sequence identity and hosts was observed in the *T. mutans* clade. ASV26, 42 and 60 were buffalo specific, and ASV3, 5, 16 were detected in both buffalo and cattle. All of them had more than 99.5% identity to the reference sequences of *T. mutans* (Fig. 2, Additional file 1: Table S1, Table 3).

Interestingly, most of the observed ASVs in the *T. parva* clade were detected only in buffalo except ASV15 (*T. parva*) and ASV11 (*Theileria* sp. buffalo), which were detected in both buffalo and cattle (Na032 and Na142, respectively) (Additional file 1: Table S1). ASV25 showed 100% identity to *Theileria* sp. *bougasvlei* but was adjacent to the *T. parva* clade (Table 3, Fig. 2).

*T. taurotragi* was detected in four cattle. ASV46 and 62 had > 99.8% identity to a *T. taurotragi* reference sequence but ASV35 had 97.8% identity, implying this can be categorized in a different genotype (Table 3).

There were two additional clades in *Theileria* (Fig. 2). One consisted of ASV8, 9, 14, 23, 49 and 85. It was almost exclusively detected in hartebeest even though ASV8 and 23 were also detected in a wild dog (Da082). They showed high identity to unspecified *Theileria* spp. The other consisted of ASV2, 47 and 106, which were detected in impala. ASV4 was detected in both hartebeest and sable antelope but also found in a buffalo (Da109). ASV12 and 13 were detected in sitatunga.

The *Babesia* clade consisted of a subclade for *B. bigemina* and *B. occultans*. ASVs in the *B. bigemina* subclade showed more than 99.8% identity to *B. bigemina* reference sequences. Both *B. bigemina* and *B. occultans* were detected only in cattle (Fig. 2, Table 3, Additional file 1: Table S1).

A *Hepatozoon canis* sequence was detected in a lion and another *Hepatozoon* sp. was detected in a wild dog. Interestingly, a *Colpodellidae* sequence, ASV57, was also detected in cattle (Fig. 2, Additional file 1: Table S1).

## Discussion

We applied the deep amplicon sequencing method to investigate piroplasmas [17, 21] in the wildlife and cattle population of the Kafue National Park and surrounding wildlife-livestock interface area of the greater Kafue ecosystem. Blood samples from 253 wild animals consisting of 12 mammalian species and 232 cattle were collected in 2018 and 2019, respectively. Although the wildlife and cattle were sampled in different but consecutive years, this may not have affected the comparison and interpretation of the results obtained, because fundamental change of biodiversity in a functional and resilient ecosystem like Kafue takes a long period.

Our data show that 45 ASVs and 23 species consisting of 4 genera (*Babesia*, *Theileria*, *Hepatozoon* and *Colpodella*) were detected. Among the 45 ASVs, 14 were identical to previously published sequences. However, 28 ASVs demonstrated percentage identity of 95.7–99.8%, suggesting that novel genotypes may also exist. ASV2, 47 and 106 presented 95.7–95.9% identity to *Theileria* sp., suggesting possible novel *Theileria* spp. or undeposited sequences of known *Theileria* spp. (asterisk in Table 3).

Within *Theileria* species, 36 ASVs were detected (Table 4). As an important natural reservoir host, buffalo had a diversity of 18 *Theileria* ASVs, which was the highest compared to other wildlife species. This finding is consistent with a previous report from a serological study involving buffalos [13]. Importantly, three ASVs of *T. parva* (OTU23 comprising ASV15, 86 and 101) were obtained from buffalo, providing important epidemiological insight into cattle in the area in terms of corridor disease transmission. Indeed, *T. parva* ASV15 was detected in cattle (Na032), suggesting possible spillover of *T. parva* from buffalo to domestic cattle. The presence of *Theileria* sp. (buffalo) (49.1%; 26 of 53) and *Theileria* sp. (bougasvlei) (18.9%; 10 of 53) in buffalo (Table 4) is of diagnostic importance as it affects the accurate detection of *T. parva* in mixed infections when performing hybridization PCR assay [33]. In addition to buffalo, *Theileria* sp. (buffalo) was also detected in the cattle population (0.4%; 1 of 232). This result supports the observations and findings from studies conducted in Kenya that also identified *Theileria* sp. (buffalo) from cattle, suggesting that *Theileria* sp. (buffalo) infection in cattle occurs in the field where buffalo and cattle share pasture [20, 34]. Nevertheless, more knowledge on the infection epidemiology and pathogenicity to cattle will be required. The presence of *T. taurotragi* circulating in the cattle population is consistent with findings in other similar studies [35]. The characterization of the parasite community and molecular ecology raises awareness about the consequences and limitations of specific diagnostic tests and requires further caution for the interpretation of the results used for diagnostics or surveillance in a specified area.

**Table 4:** Prevalence of species detected in the sampled wildlife species and cattle from the Kafue ecosystem

Parasitic species	Impala	Hartebeest	Sable	Sitatunga	Lion	Wild dog	Buffalo	Cattle	Corresponding ASV
*T. velifera*	1 (0.9%)						26 (49.1%)	136 (58.6%)	1, 6, 7, 29, 55, 64, 92
*T. mutans*							20 (37.7%)	140 (60.3%)	3, 5, 16, 26, 42, 60
*T. parva*							8 (15.1%)	1 (0.4%)	15, 86, 101
*T. taurotragi*								4 (1.7%)	35, 46, 62
*Theileria *sp. (buffalo)							26 (49.1%)	1 (0.4%)	10,11,18,22
*Theileria *sp. (bougasvlei)							10 (18.9%)		25
*Theileria* sp. (sable)		15 (31.9%)				1 (50.0%)			8,14,85
*Theileria *sp. (waterbuck)		12 (25.5%)	8 (100.0%)				1 (1.9%)		4
*Theileria *sp. (tsessebe)		8 (17.0%)				1 (50.0%)			9,23,49
*Theileria *sp. (giraffe)	65 (61.3%)								2,47,106
*T. bongo*				3 (75.0%)					12, 13
*B. bigemina*								24 (10.3%)	19, 32, 67, 71
*Babesia *sp.						1 (50.0%)			58
*B. occultans*								4 (1.7%)	28
*H. canis*					1 (25.0%)				40
*Hepatozoon* sp.						1 (50.0%)			82
*Colpodellodae*								1 (0.4%)	57
Total head sampled	106	47	8	4	4	2	53	232	

*ASVs* amplicon sequence variants

*Babesia* was predominantly observed in cattle but also detected in wild dogs. *Babesia bigemina* (10.3%; 24 of 232) and *B. occultans* (1.7%; 4 of 232) were the only species detected in cattle (Table 4), of which *B. bigemina* is a pathogenic parasite to cattle causing the clinical disorder of babesiosis, also known as redwater. These findings are similar to other comparable studies in southern Africa where the presence of *Babesia* in cattle and wild animals, particularly buffalo, was assessed [36]. To the best of our knowledge, this is the first report of the non-pathogenic *B. ocultans* in Zambia. However, its specific vectors, impact on cattle, diagnostic consequence in *Babesia* mixed infection and implication of infection to wildlife are not evaluated.

Despite not being classified in the order of piroplasmida but Eucoccidiorida, Apicomplexan species of *Hepatozoon canis* and *Hepatozoon* sp. were detected in African lion and wild dog samples, respectively. Divergent from other arthropod-borne parasites transmitted through the vector’s salivary glands at the time of feeding, *Hepatozoon* are transmitted to the carnivore host exclusively by ingestion of infected vectors (ticks) during grooming [37, 38]. They cause subclinical infection in wild carnivores and clinical infection in domestic dogs [39]. Previous studies on free-ranging wild carnivores in Zambia have indicated the widespread presence of *Hepatozoon* sp. in lions [40]. This highlights the considered epidemiological role of wild carnivores as a sylvatic reservoir of *Hepatozoon* and presents the risks of likely spillover of *Hepatozoon* infections to domestic carnivores in the interface area.

Genera of *Colpodella* are part of Alveolata organisms that are originally known to be free-living. However, recent studies have revealed the parasitic nature of *Colpodella* sp. as a human erythrocyte parasite (HEP) that has lately been reported from China to cause relapsing fevers and neurological symptoms in humans [41, 42]. Furthermore, the detection of *Colpodella* sp. in ticks suggests that this parasite may potentially be transmitted by tick vector(s) [41]. We detected a *Colpodella* sequence from one of the cattle samples, with the sequence identity of 79.6% with the reported human cases (GQ411073; *Colpodella* sp. HEP). The sequence detected from our cattle sample showed a perfect match (100% identity) to GenBank MN103986 (Colpodellidae clone PL31), reported in raccoon dog in Poland [43]. Thus, the detection of *Colpodella* sp. from a cattle sample implies support of those findings that some of the *Colpodella* species are associated with vertebrates and possibly cause disease. What vector is involved, how the parasite is maintained and the risk that the cattle may pose for human infection are largely undetermined. It would be important to determine the zoonotic scale of *Colpodella* infection to rule out incidental infections.

Identification of multiple infection is the another advantage of deep amplicon sequencing [21]. It is known that the African buffalo is simultaneously infected with multiple species of *Theileria* [44]. According to our study, the African buffalo is the most infected animal with multiple species of *Theileria* (see Fig. 2 and additional data; Additional file 1: Table S1). Besides, most of the cattle were also co-infected with *Theileria velifera* and *Theileria mutans*. It is reported that co-infection of multiple *Theileria* spp. in cattle results in dramatically different pathological disorders compared to single-species infections [45]. Further studies with expanded sample size might demonstrate similar interactions in wildlife as well. This is particularly important since Zambia’s cattle population stronghold is in the Itezhi-Tezhi district which is adjacent to the KNP. This is cardinal as accurate diagnosis and effective control (vaccinations) of piroplasm parasites need to take the parasite community data into account. Hartebeest also tended to be co-infected with *Theileria* spp. In contrast, the impalas were mainly infected with *Theileria* spp. isolated from giraffe but hardly co-infected with other piroplasmas.

The identification of tick-borne pathogens in the wildlife and cattle population in the study area supports the apparent presence of the known tick vectors implicated in their transmission. Particular *Theileria* species are known to be transmitted by specific ixodid tick species of *Rhipicephalus appendiculatus*, *R. zambeziensis* and *Amblyomma variegatum*, while *Babesia* species are transmitted by *R. microplus*, *R. decororatus* and *R. evertsi* [46, 47]*.* The tick species associated with transmission of *Hepatozoon* species is the *Rhipicephalus sanguineus* sensu lato (s.l.) [48]. To identify the unknown vectors of some of the parasites described in this study, there is need to conduct tick piroplasm metagenomic analysis. This would further illustrate the piroplasm parasite ecocycle more precisely.

## Conclusion

Molecular epidemiology based on the strength of knowledge of the cryptic parasite community and diversity is essential in the control of piroplasmosis. Mapping of the piroplasm parasites in all major livestock landscapes beyond the Kafue ecosystem using the metagenomic approach may benefit piroplasmosis control in Zambia through high-resolution data to precisely guide diagnosis, vaccination and movement controls.

## Supplementary information

**Additional file 1.** Metadata from Illumina MiSeq sequencing and ASV analysis.

## Data Availability

Data supporting the conclusions of this article are included within the article and its additional file. Representative ASV sequences were deposited in the GenBank database under the accession numbers MT814722–MT814766.
